# When the mask slips: A peripheral T-cell lymphoma disguised as lupus with myelofibrosis in a patient with May-Hegglin syndrome

**DOI:** 10.1016/j.lrr.2024.100498

**Published:** 2024-12-20

**Authors:** V Da Silva Constante, H Couvert, A Wolfromm, M Ilzkovitz

**Affiliations:** a Hematology Department, Institut Jules Bordet – Hôpital Universitaire de Bruxelles (H.U.B.), Université Libre de Bruxelles (ULB), Brussels, Belgium; b Internal Medicine Department, Institut Jules Bordet – Hôpital Universitaire de Bruxelles (H.U.B.), Université Libre de Bruxelles (ULB), Brussels, Belgium

**Keywords:** Peripheral T-cell lymphoma, PTCL, NOS, Myelofibrosis, Systemic lupus erythematosus, May-Hegglin syndrome

## Abstract

We describe the case of a female patient with May-Hegglin syndrome who developed peripheral T-cell lymphoma not otherwise specified. The patient presents with systemic lupus erythematous phenotype and myelofibrosis secondary to T-cell lymphoma. Peripheral T-cell lymphoma not otherwise specified, represents 25 % of all peripheral T-cell lymphoma. Its diagnosis remains challenging due to the polymorphous clinical presentation and pathological heterogeneity. Myelofibrosis associated with malignant lymphomas is rare and peripheral T-cell lymphoma is even rarer. To our knowledge, this is the first case to describe an association between May-Hegglin syndrome and a peripheral T-cell lymphoma.

## 1 Case description

A 66-year-old female patient visited her general practitioner with complaints of diffuse arthralgia, anorexia leading to a 5 kg weight loss in 2 months, slight dyspnea, non-productive cough, and epistaxis. She was diagnosed at the age of 10-year-old with May-Hegglin syndrome, a constitutional thrombopathy, complicated by platelets and red blood cell alloimmunization due to repeated transfusions. Her average platelet level was 40 000/µL. In 2007, she was diagnosed with lung adenocarcinoma and was treated with lobectomy. She underwent radiotherapy for lymph node recurrence in 2009.

Upon admission to the emergency department, the patient mentioned having taken multiple trips to the Caribbean in recent years, with her most recent travel being to the Seychelles five months ago. By reviewing her treatment history, the patient only took Ezetimibe 10 mg1x/d, Montelukast 10 mg 1x/d, Atrovent, Trelegy 92/55/22 mcg 1x/d, Nexiam 20 mg 1x/d, Diazepam 5 mg 1x/d. Thus, no exposure to haematotoxic substances was found.

The clinical examination revealed widespread swollen lymph nodes, bronchial crackles, a petechial rash, and an aortic murmur. There was no synovitis.

Blood tests revealed pancytopenia with marked thrombocytopenia at 4 000/µL (N 150 000–440 000/µL), normocytic anemia with hemoglobin at 7.3 g/dL (13.0–18.0 g/dL), and leukopenia with neutropenia at 440/µL (N 1 500–6 700/µL).

Immune thrombocytopenic purpura was suspected due to the viral-like illness and severe decrease in platelet count. Intravenous immunoglobulin (IVIg) at 1 g/kg was administered twice. However, thrombocytopenia did not improve, raising the suspicion of an underlying secondary etiology.

Paroxysmal nocturnal hemoglobinuria clone mutation was absent. A bone marrow aspiration revealed a low cellularity without excess blasts and a relative excess in plasma cells and lymphocytes. Blood and bone marrow immunophenotypes confirmed the polyclonal nature of the plasma cells and found no abnormalities in T- or B-lymphocytes. Extensive infectious workup came back negative (Table 1).

**Table 1:** Infectious work-up.

Site	Type of assessment	Pathogen	Results
Blood	Serology	EBV	VCA-IgG positive VCA-IgM negative
		CMV	IgG positive IgM negative
		HBV	Ag Hbs negative Anti-Hbs positive
		HCV	IgG negative
		Parvovirus B19	IgM negative IgG negative
		Toxoplasma gondii	IgM negative IgG positive
		Brucella	Rose Bengale negative
		Coxiella burnetii	IgG negative
		Bartonella Henselae	IgG/IgM negative
		Leishmania	IgG negative
	PCR	Parvovirus B19	Negative
		EBV	Positive: 2 500 copies
		CMV	Negative
	Quantiferon		Negative
Lymph node	PCR	EBV	Positive
Bone marrow	PCR	Leishmania	Negative

CMV, Cytomegalovirus; EBV, Epstein - Barr virus; HBV, Hepatitis B virus; HCV, Hepatitis C virus; PCR, Polymerase Chain Reaction.

**Fig. 1:**
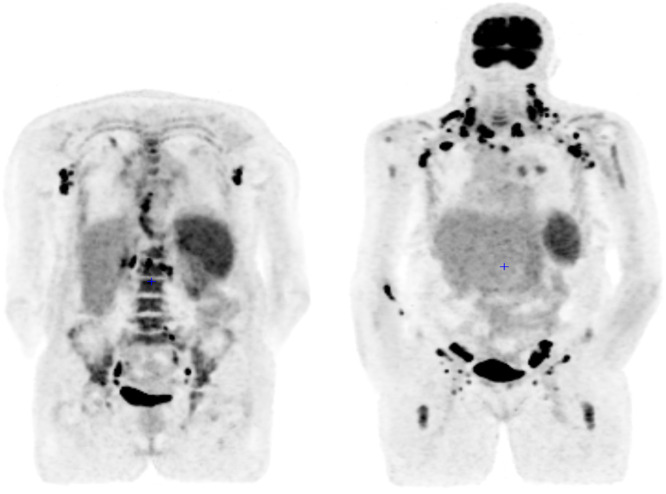
Positron emission computed tomography with ^18^F-fluorodeoxyglucose.

^18^F-fluorodeoxyglucose positron emission computed tomography revealed hypermetabolic lymph nodes (SUVmax 18.6), bone marrow, pleuropulmonary, and splenic involvements highly suggestive of lymphoma (Fig. 1).

Despite receiving treatment with a thrombopoietin receptor agonist (TPOa), the patient's thrombocytopenia continued to worsen until the platelet count reached 0/µL, which made it impossible to conduct bone marrow and lymph node biopsies. The patient ultimately developed febrile neutropenia with respiratory symptoms and was empirically treated with Ceftazidim and Vancomycin for seven days.

Given the atypical clinical and laboratory findings, an autoimmune assessment was performed, revealing lupus-like serology with FAN at 1/1280, positive anti-double-stranded DNA antibodies at 43 U/mL (*N*< 30 U/mL), and positive anti-nucleosome antibodies at 172 U/mL (*N*< 20 U/mL).

**Fig. 2:**
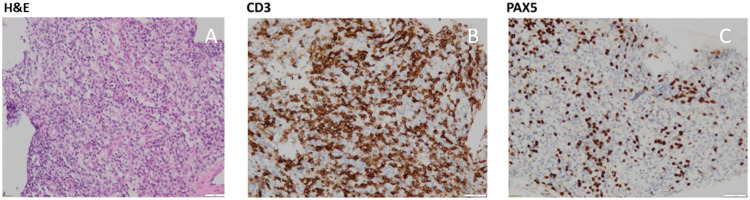
Lymph node biopsy: Immunohistochemistry with haematoxyline-eosine (H&E) staining positive cells (A). Immunohistochemistry with CD3 staining positive cells (B). Immunohistochemistry with PAX5 staining positive cells (C). Original magnification x 10.

However, the possibility of Systemic Lupus Erythematosus (SLE) was considered unusual given the patient's age, clinical presentation, and absence of a family history of autoimmunity. A review of lupus mimickers has therefore been explored and the suspicion of hematological malignancies arose. Despite severe thrombocytopenia, a lymph node biopsy was performed. Pathological analysis revealed a predominantly lympho-histiocytic population, associated with rare eosinophils. Regarding immunostaining, anti-CD20, and anti-PAX5 showed the presence of a few typical B lymphocytes, anti-CD30 showed no Hodgkin cells, and anti-CD3 confirmed the presence of a majority of T lymphocytes, some of which were discreetly atypical. Anti-CD4, anti-PD1, anti-CD10, anti-BCL6, and anti-CXCL13 immunostainings were non-contributory due to a lack of residual material (Fig. 2). Epstein-Barr virus encoding region (EBER) in situ hybridization was positive. The lymph node NGS panel was positive for two distinct TET2 mutations. Monoclonal T-cell receptor (TCR) gene rearrangement was found in the blood.

Following lymph node biopsy, steroids were introduced and TPOa was pursued. The patient received methylprednisolone at a dose of 80 mg/d for five days, resulting in a significant improvement in thrombocytopenia and leukopenia but did not improve anemia. Five days after stopping the corticosteroid, cytopenia worsened and the patient developed a new episode of febrile neutropenia leading to the intensive care unit for septic shock.

After returning from intensive care, the patient reported retrosternal chest pain, due to pericardial effusion likely due to SLE. In this context, she was treated with colchicine 1 mg/d for 6 days, but due to poor digestive tolerance, she was switched to corticosteroids.

**Fig. 3:**
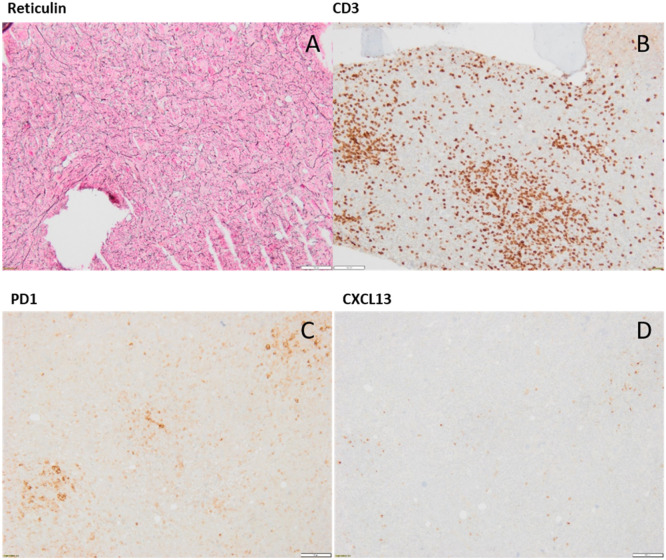
Bone marrow histology: Immunohistochemistry with reticuline staining (A). Immunohistochemistry with CD3 staining positive cells (B) Immunohistochemistry with PD1 staining positive cells (C). Immunohistochemistry with CXCL13 staining positive cells (D). Original magnification x 4.

Bone marrow biopsy was ultimately performed during platelet transfusion. Histology revealed the presence of grade 2 reticulinic myelofibrosis with polyclonal T-cell lymphocytosis (Fig. 3). Classical mutation panels for primary myelofibrosis (JAK2, CAL-R, and MPL) and myeloid mutation panels (including TET2) were not detected. The karyotype was normal.

Finally, a diagnosis of Peripheral T-cell Lymphoma Not Otherwise Specified (PTCL, NOS), with an SLE-like phenotype associated with reactive myelofibrosis was retained based on monoclonal TCR gene rearrangement performed on blood and lymph nodes and classical TET2 gene mutation.

The patient was then treated with a reduced dose of Cyclophosphamide-Doxorubicin-Vincristine-Prednisone (mini-CHOP). Her general condition and cytopenias rapidly improved under chemotherapy.

## 2 Discussion

We describe a singular case of PTCL, NOS associated with a lupus-like phenotype and myelofibrosis. Accounting for 25 % of all PTCL, it is the most prevalent type of PTCL around the age of 60. Due to the polymorphous clinical presentation, pathological heterogeneity, and broad molecular spectrum, PTCL diagnosis remains challenging. Although nodal involvement is common at diagnosis, any organ can be affected, including bone marrow, spleen, and liver [1]. Typical laboratory findings include inflammatory syndrome, elevated lactate dehydrogenase, polyclonal hyper-gammaglobulinemia, anemia, and lymphopenia. [2].

PTCL is characterized by the clonal expansion of T cells and can be classified into various subtypes, including Angioimmunoblastic T-cell Lymphoma (AITL), based on unique morphological, immunophenotypic, molecular, and clinical features. PTCL, NOS diagnosis is based on the exclusion or lack of argument for other specific PTCL subtypes. Recent progress in histology, molecular analysis, and classification systems has led to the reclassification of approximately 15 % of PTCL, NOS cases as AITL [1,3].

Differential diagnosis between PTCL, NOS and AITL is usually based on lymph node histology, but the recent contribution of molecular biology broadens the diagnostic options. Pathological assessment through immunostainings is essential in the differential diagnosis. On one hand, typical positive staining for CD4 predominance, CD30, CD56, frequent antigen loss (CD5, CD7), and cytotoxic granules in PTCL, NOS. On the other hand, CD4, CD10, BCL6, PD1, CXCL13, and ICOS, with a loss of CD7 in AITL. Moreover, up to 80 % of AITL are also EBER positive [4,5]. Bone marrow involvement is another feature of PTCL, often characterized by atypical lymphocytosis. Some degrees of bone marrow fibrosis are rarely reported. Monoclonal TCR gene rearrangement is not uncommon in PTCL and is useful in supporting the diagnosis of T-cell lymphoma. In PTCL, NOS and AITL, clonal rearrangements of TCR genes are reported in >80 % of cases. Genetic mutations may also be present in bone marrow or lymph nodes in up to 50 % of AITL patients. Mutations are found in epigenetic regulator genes, like TET2, IDH2, or DNMT3A, or in small GTPases, like RhoA in cases of PTCL, NOS and AITL [1,3,6].

In this case, the SLE phenotype and genetic tests might support a diagnosis of AITL. However, the absence of specific immunohistochemical characteristics necessitates reclassification as PTCL-NOS. SLE was the primary diagnostic hypothesis in this case. The patient exhibited features compatible with both SLE and AITL, such as polyadenopathy, cytopenias, pericarditis, and arthralgia, which are common in SLE. Despite not being used for diagnosis, the patient fulfilled the 2019 ACR/EULAR SLE classification criteria with a score of 39, strongly suggestive of SLE [7].

SLE is a chronic autoimmune disease that can affect nearly every organ. Many diseases share clinical and biological similarities with SLE, including infectious disorders, Kikuchi's disease, Castleman's disease, autoimmune lymphoproliferative syndrome, and AITL [2]. However, those differential diagnoses were considered less likely compared to AITL. B symptoms, cytopenias, and polyadenopathy can mimic those of hematologic malignancies. These signs and symptoms are more often seen as SLE progresses, rather than at the initial presentation, which can lead to misdiagnosis. In SLE, chronic autoimmune stimulation can increase the risk of secondary malignancies, with non-Hodgkin lymphoma, specifically diffuse large B cell lymphoma, being the most common hematologic cancer associated with the disease [[8], [9], [10], [11]].

Although rare, an association between AITL and SLE has been reported. In 2023, Wang et al. documented seven cases of AITL resembling SLE, involving three men and four women with an average age of 55 [12]. The underlying mechanism of the autoimmune manifestations associated with AITL is not fully understood. However, it is hypothesized that the altered function of the follicular T helper cells may contribute to B-cell survival and antibody production dysregulation [13].

Another notable aspect of this case is the presence of myelofibrosis. Myelofibrosis can be either primary or secondary. Primary myelofibrosis is a clonal myeloproliferative disorder characterized by fibrous tissue deposits in the bone marrow, commonly associated with JAK2, CALR, or MPL mutations [14,15]. Secondary myelofibrosis typically develops after essential thrombocythemia or Vaquez polycythemia. Myelofibrosis associated with malignant lymphomas is rare. The pathophysiology remains unclear, but a lymphomatous infiltration is usually suggested [16]. Among lymphomas associated with myelofibrosis, AITL is infrequently reported [17,18]. In 2015, Sekiguchi et al. reported only fifteen cases of T-cell lymphoma, including seven cases of AITL and two cases of PTCL, NOS complicated with myelofibrosis. Since then, only two more cases, counting ours, have been reported [20]. Various treatments were used for T-cell lymphoma, but only 50 % of patients showed improvement, indicating that these treatments are not consistently effective for managing MF and require careful monitoring. Furthermore, seven patients died within two years of diagnosis, highlighting the disease's extremely poor prognosis [19].

Given that the patient in our case met the criteria for SLE, it is important to consider autoimmune myelofibrosis (AIMF). In 2003, Pullarkat et al. first described primary AIMF in patients with markers of autoimmunity but without a well-defined autoimmune disease (AID) [14]. On the other hand, "secondary" AIMF refers to myelofibrosis occurring with a well-defined AID, such as SLE, or less commonly, scleroderma and Sjögren's syndrome [14,15,21].

As discussed earlier, myelofibrosis in this case could be attributed to either lymphomatous infiltration or autoimmune mechanisms. The patient's response to chemotherapy supports the hypothesis of a lymphomatous cause.

Lastly, to our knowledge, this is the first case describing an association between May-Hegglin syndrome and a PTCL.

## 3 Conclusion

The diagnosis of PTCL, and its subtypes, can be challenging especially when associated with myelofibrosis and a lupus-like phenotype in the setting of severe thrombocytopenia.

## Informed consent

Written informed consent was obtained from the patient, including permission for the publication of the image.

## CRediT authorship contribution statement

**V Da Silva Constante:** Writing – original draft, Data curation. **H Couvert:** Writing – original draft, Data curation. **A Wolfromm:** Writing – review & editing, Supervision, Conceptualization. **M Ilzkovitz:** Writing – review & editing, Validation, Supervision, Methodology, Conceptualization.

## Declaration of competing interest

The authors declare that they have no known competing financial interests or personal relationships that could have appeared to influence the work reported in this paper.
